# Simultaneous Protective Immune Responses of Ducks against Duck Plague and Fowl Cholera by Recombinant Duck Enteritis Virus Vector Expressing *Pasteurella multocida* OmpH Gene

**DOI:** 10.3390/vaccines10081358

**Published:** 2022-08-19

**Authors:** Nisachon Apinda, Anucha Muenthaisong, Paweena Chomjit, Kanokwan Sangkakam, Boondarika Nambooppha, Amarin Rittipornlertrak, Pongpisid Koonyosying, Yongxiu Yao, Venugopal Nair, Nattawooti Sthitmatee

**Affiliations:** 1 Department of Veterinary Biosciences and Veterinary Public Health, Faculty of Veterinary Medicine, Chiang Mai University, Chiang Mai 50100, Thailand; 2 Department of Food Animal Clinic, Faculty of Veterinary Medicine, Chiang Mai University, Chiang Mai 50100, Thailand; 3 The Pirbright Institute, Ash Road, Pirbright, Woking GU24 0NF, UK; 4 Jenner Institute, University of Oxford, Oxford OX1 2JD, UK; 5 Department of Zoology, University of Oxford, 11a Mansfield Road, Oxford OX1 3SZ, UK; 6 Excellence Center in Veterinary Bioscience, Chiang Mai University, Chiang Mai 50100, Thailand

**Keywords:** duck plague, fowl cholera, viral vector, recombinant vaccine, CRISPR/Cas 9

## Abstract

Duck enteritis virus and *Pasteurella multocida* are major duck pathogens that induce duck plague and fowl cholera, respectively, in ducks and other waterfowl populations, leading to high levels of morbidity and mortality. Immunization with live attenuated DEV vaccine containing *P. multocida* outer membrane protein H (OmpH) can provide the most effective protection against these two infectious diseases in ducks. We have recently reported the construction of recombinant DEV expressing *P. multocida* ompH gene using the CRISPR/Cas9 gene editing strategy with the goal of using it as a bivalent vaccine that can simultaneously protect against both infections. Here we describe the findings of our investigation into the systemic immune responses, potency and clinical protection induced by the two recombinant DEV-ompH vaccine constructs, where one copy each of the ompH gene was inserted into the DEV genome at the UL55-LORF11 and UL44-44.5 intergenic regions, respectively. Our study demonstrated that the insertion of the ompH gene exerted no adverse effect on the DEV parental virus. Moreover, ducklings immunized with the rDEV-ompH-UL55 and rDEV-ompH-UL44 vaccines induced promising levels of *P. multocida* OmpH-specific as well as DEV-specific antibodies and were completely protected from both diseases. Analysis of the humoral and cellular immunity confirmed the immunogenicity of both recombinant vaccines, which provided strong immune responses against DEV and *P. multocida*. This study not only provides insights into understanding the immune responses of ducks to recombinant DEV-ompH vaccines but also demonstrates the potential for simultaneous prevention of viral and bacterial infections using viral vectors expressing bacterial immunogens.

## 1. Introduction

Fowl cholera is a contagious bacterial disease of all types of birds. It is caused by *Pasteurella multocida* and is the leading cause of huge economic loss in the poultry industry world-wide [1,2]. The major membrane protein of the *P. multocida* envelope, the outer membrane protein H (OmpH), is a cross-protective antigen highly conserved among avian *P. multocida* strains. When incorporated as a fowl cholera vaccine candidate, it provided strong protective immunity against *P. multocida* in chickens [3,4,5,6,7] and ducks [8,9].

Duck viral enteritis, also called duck plague (DP), is an acute contagious disease of all ages and species among Anseriformes (ducks, geese, and swans). Caused by duck enteritis virus (DEV), a member of the family Herpesviridae, the disease is associated with high morbidity and mortality [10,11]. Immunosuppressive effects of DEV also result in secondary bacterial infections from pathogens such as *Pasteurella multocida, Riemerella anatipestifer* and *Escherichia coli,* as has often been observed in many natural outbreaks [12].

Vaccinations are the cornerstones for the control of diseases caused by many avian pathogens, including DEV. In 1963, Jansen et al. first found the attenuated DEV vaccine could protect ducks against DEV challenge, and since then, this type of vaccine has been used extensively worldwide [13]. Currently, commercial vaccines for the prevention of both duck viral enteritis and fowl cholera are routinely used in duck production in areas where these diseases are a major threat. All of these are monovalent vaccines, and some require multiple immunizations for full efficacy. Multiple injections of vaccines lead to increased stress, resulting in compromised immunity and reduced egg production [14]. Moreover, repeated administration of monovalent vaccines is costly, time-consuming, and labour-intensive, making it a major limitation to these high-growth industries [15]. Because of these limitations, there is a strong need for new innovative approaches in vaccination-based control strategies to protect against avian diseases in traditional and industrial duck farming.

The study of viral-vector vaccines has expanded rapidly in recent years, following an improved knowledge of viral biology and immunology. Recently, the promising innovation of gene-editing technology based on clustered regularly interspaced short palindromic repeats (CRISPR) and CRISPR-associated 9 (Cas9) have been well developed and extensively applied for genomic editing in veterinary biological research [16,17]. The method provides a novel platform in the development of recombinant viral vaccines because it has been demonstrated as a more fast, powerful, simple, efficient, and straightforward approach for editing viral genomes [16,18,19]. At present, DEV has been successfully modified and used as live viral vectors as a recombinant vaccine to provide rapid protection against multiple poultry viruses based on using CRISPR/Cas9 strategy [20,21,22,23,24,25,26]. In our previous report, we successfully developed DEV-ompH vectored vaccine candidates, rDEV-ompH-UL55 and rDEV-ompH-UL44, by inserting the ompH gene of *P. multocida* into each UL55-LORF11 and UL44-44.5 intergenic region of the DEV genome of the vaccine (jansen) strain, respectively, using NHEJ-CRISPR/Cas 9 with the Cre-Lox system. Our data indicate that CRISPR/Cas9 is a promising strategy for screening multiple gene loci in the DEV genome as a stable insertion site of foreign genes in mammalian cells. Moreover, the presence and the expression of the ompH gene were successfully detected by PCR, immunofluorescence and Western blot analysis for both rDEV-ompH-UL55 and rDEV-ompH-UL44 in chicken embryo fibroblasts (CEFs), and the insert could be stably maintained in the recombinant viruses for 15 passages without any adverse effects on viral replication [27].

Cellular and humoral immune responses are important in resisting and clearing viral infections [28]. We investigated whether the two recombinant rDEV-ompH-UL55 and rDEV-ompH-UL44 viruses could be used as a bivalent vaccine capable of inducing simultaneous protection against the two major viral and bacterial pathogens in duck hosts. For this, we characterized both the humoral and cellular immune responses after immunization of ducks with rDEV-ompH-UL55 and rDEV-ompH-UL44 viruses. Furthermore, the protective efficacy of the two recombinant viruses against the lethal DEV and *P. multocida* strain X-73 challenge in ducks was also examined.

## 2. Materials and Methods

### 2.1. Viruses and Cells

The DEV Jansen strain, obtained from the Bureau of Veterinary Biologics Department of Livestock Development, Ministry of Agriculture and Cooperative Thailand, was used for the construction of the DEV recombinant candidate and DEV wild type. The recombinant DEV-ompH of UL55-LORF 11 and DEV-ompH of UL44-44.5 were constructed by inserting the ompH gene of *P. multocida* via NHEJ-CRISPR/Cas9 and Cre-Lox system as described previously [27]. The viruses were propagated in primary chick embryo fibroblasts (CEF) prepared from 10-day-old specific-pathogen-free embryonated chicken eggs. CEF cells were maintained with M199 medium (Thermo Fisher Scientific, Waltham, MA, USA), supplemented with 5% fetal bovine serum (FBS, Sigma-Aldrich, St. Louis, MO, USA), 100 units/mL of penicillin and streptomycin (Thermo Fisher Scientific), and 0.25 mg/mL Fungizone (St. Louis, MO, USA) under a 5% CO_2_ atmosphere. The supernatant of virus-infected cells was collected and stored at −80 °C until use. The local virulent strain of DEV was used for the challenge.

### 2.2. Immunization and Challenge Exposure

The project underwent an ethical review and was given approval by an institutional animal care and use committee. The Animal Welfare Committee of the Faculty of Veterinary Medicine, Chiang Mai University, oversaw the use of the laboratory animals in accordance with laboratory animal ethics protocols (license number S28/2562). All experiments followed the guidelines that had been designated by the Guide for the Care and Use of Agricultural Animals in Research and Teaching (the Ag Guide, FASS 2010).

A total of 100 3-week-old DEV and *P. multocida* seronegative Khaki Campbell ducks (*Anas platyrhynchos*: APA Farm Co., Ltd., Chiang Mai, Thailand) were divided into 5 groups, with 20 ducks in each group. The ducks were injected intramuscularly with different vaccines presented in Table 1. Briefly, each group was administered with 10^5^ PFU (a recommended dose for the DEV vaccine) of rDEV-ompH-UL55, rDEV-ompH-UL44, 10^3.5^ PFU of DEV-WT vaccine (recommended dose from the Department of Livestock Development), and 100 µg of rOmpH + montanide (SEPPIC, Paris, France) and ducks in the control group were inoculated with PBS. Ducks were boosted with the same dose of vaccine four weeks after the first vaccination. All duck sera were collected from blood collection every two weeks post-immunization for the serological responses against DEV and *P. multocida* using ELISA by the method described previously [8,29]. After the second booster dose, each group of ducks was randomly subdivided into two groups (10 per group) for challenge exposure to virulent DEV or *P. multocida*, respectively. Ducks were challenged with a 100-fold 50% duck lethal dose (DLD_50_) of local virulent strain DEV and 3.5 × 10^3^ CFU/mL of *P. multocida* strain X-73 (ATCC#11039, provided by Professor Dr. Takuo Sawada, Nippon Veterinary and Life Science University, Japan) by intramuscular injection at 4 weeks after the booster immunization. Ducks were observed daily for the clinical signs of the diseases for 1 week. Tissues of liver, spleen, and lung of euthanized ducks in each group were collected, and the viral load was confirmed by RT-PCR or *P. multocida* bacterial isolation.

### 2.3. Quantitative Real-Time Polymerase Chain Reaction (RT-PCR)

To determine the number of viruses sustained in organs infected with rDEV-ompH of UL55 and rDEV-ompH of UL44 or DEV-WT, we intramuscularly inoculated three groups of ducks (three per group) with 10^5^ PFU of each DEV recombinant virus or DEV wild type vaccine. All ducks in the three groups were humanely euthanized on day 7 post-challenge (pc) following the AVMA guidelines for the euthanasia of animals from 2020 [30], and their organs, including liver, spleen, and lungs, were obtained to determine virus titers using a one-step real-time TaqMan RT-PCR assay modified from a previous study [31]. Briefly, The RT-PCR primers were designed based on the sequence of the gI gene: the RT-gI-F primer is (5′-GCCGTGGAAGACAGAC-3′), and the RT-gI-R primer is (5′-CCAAGACGAGGGCAATCA-3′). The primers were checked by running a conventional PCR. The standard curves of the real-time PCR were generated by successive dilutions of recombinant plasmid pCR2.1-TOPO-gI, which was constructed by amplifying the fragment of gI with primer pair DEV-gI-F and DEV-gI-R and cloning into the TOPO vector (Invitrogen, Waltham, MA, USA) following the manufacturer’s instructions. The real-time amplifications were carried out in a 96-well plate in a 20 μL reaction volume containing 10 μL of SensiFAST SYBR Lo-ROX Master Mix (Bioline, Meridian Bioscience, London, UK), 0.5 μL each of forward and reverse primers and 1 μL of the 1:10 diluted recombinant plasmid. The temperature profile for SYBR Green CFX96 Real-Time PCR (Bio-Rad Laboratories, Hercules, CA, USA) was 95 °C for 1 min, followed by 45 cycles of 95 °C for 5 s, 60 °C for 20 s and 72 °C for 25 s. Each sample had 3 replicates; both negative control and blank control were run along with the samples. After an SYBR Green CFX96 Real-Time PCR run, data acquisition and each cycle threshold (Proctor) value analyses were determined using the CFX Maestro System Software (Bio-Rad).

### 2.4. ELISA Test to Evaluate Immune Response to P. multocida Strain X-73

The ELISA for duck antibody was performed following a previous study [9]. Briefly, 96-well plates were coated with 100 μL of whole cells of *P. multocida* strain X-73 (approximately 10^4^ CFU/well) and incubated overnight at 4 °C. After being washed three times with a washing buffer (PBS containing 0.05% Tween: PBST), the plates were blocked with 1% bovine serum albumin (BSA)-PBS for 1 h at 37 °C. The plates were then washed again, and 100 μL of 1:100 dilution of duck sera was added to the specimens in duplicate, then incubated for 1 h at 37 °C. Horseradish peroxidase (HRP)-conjugated rabbit anti-duck IgG antibody (Sigma-Aldrich) was used as the secondary antibody at 1:4000 ratios of dilution and incubated for 1 h at 37 °C. After being washed three times, 100 μL tetramethylbenzidine (TMB; KPL, Thermo Fisher Scientific) was added to each specimen. After incubation for 15 min, the reaction was terminated by the addition of 50 μL 2 N H_2_SO_4_. Optical density was measured at 450 nm (OD450) using an automatic ELISA plate reader (AccuReader; Metertech, Taipei, Taiwan).

### 2.5. ELISA to Evaluate Immune Response to DEV

The ELISA for duck antibody was performed following a previous study [8]. Each well was coated with 100 μL of attenuated DEV Jansen strain at 1:10,000 dilution of 10^3^ TCID_50_, followed by incubation at 4 °C overnight. After washing three times with 0.05% PBST washing buffer, 100 μL of blocking buffer (1% BSA-PBS) was added to each well, and the plates were kept for 1 h at 37 °C. Subsequently, after washing three times with PBST, 100 μL of individual sera at 1:800 dilution from each group was added to each well in duplicate, and plates were incubated for 1 h at 37 °C. After washing the plates three times with PBST, HRP-conjugated rabbit anti-duck IgG (Sigma-Aldrich) was used as the secondary antibody at a 1:20,000 ratio, and the specimens were incubated for 1 h at 37 °C. Finally, after three further washings, 100μL TMB diluted in substrate buffer was added to each well and incubated in the dark for 30 min at 37 °C. Subsequently, the colour reaction was stopped by adding 50 μL of 2N H_2_SO_4_. The absorbance was read at a wavelength of 450 nm. The results were expressed as OD.

### 2.6. Determination of Cellular Response by MTT Proliferation Assays

At 8 weeks post-immunization, peripheral blood mononuclear cells (PBMCs) were modified from a previous study [9]. Briefly, PBMCs were isolated from heparinized blood samples and resuspended at a concentration of 5 × 10^5^ cells per mL in RPMI 1640 (Invitrogen, USA) supplemented with 10% fetal bovine serum (FBS), 100 U/mL penicillin, and 100 μg/mL streptomycin. Subsequently, 100 μL of cell suspension were dispensed into 96-well culture plates in triplicate and were stimulated in the 5 μg/mL of purified heat extract protein of *P. multocida*, DEV 10^4^ TCID_50_ and concanavalin A (ConA; Thermo Fisher Scientific) at the final concentration of 5 μg/mL was used as a cell control. After 48 h incubation at 37 °C with 5% CO_2_, 10 μL MTT (3-(4,5-dimethylthiazol-2-yl)-2,5-diphenyltetrazolium bromide, Sigma-Aldrich) (5 mg/mL) per well was added and incubated for another 4 h. The optical density (OD) was determined in triplicate against a reagent blank at a test wavelength of 540 nm. Results were expressed as stimulation indices (SI) = mean counts per minute in stimulated wells/mean counts per minute in media wells.

### 2.7. Serum Detection Level of IFN-γ and IL-4 Cytokine Assay

Duck sera from each group were collected at 4, 6 and 8 weeks after the first immunization for the cytokine assay. To determine the levels of Th1-type cytokines and Th2-type cytokines, levels of IFN-γ and IL-4 were analyzed using commercial duck IFN-γ and IL-4 sandwich ELISA kits according to the manufacturer’s instruction (MyBioSource Inc., San Diego, CA). Moreover, serum was also collected from the surviving ducks 1 week post-challenge for IFN-γ and IL-4 measurement with the same protocol.

### 2.8. Detection of CD4+ and CD8+ T Lymphocytes in Peripheral Blood by Flow Cytometry

Blood was collected from three ducks per group for the identification of CD4 and CD8 at 4 and 8 weeks after the first immunization. PBMCs were isolated as described previously [9]. The cells were washed twice in PBS (0.01 M, pH7.4) and adjusted to a final concentration of 1 × 10^6^ cells per mL. Then indirect staining of the cells was carried out as follows: mouse anti-duck CD8 alpha monoclonal antibody and mouse anti-duck CD4 monoclonal antibody (Bio-Rad) were added onto the cells and incubated for 30 min at 4 °C in the dark, and the cells were further stained with 1:200 diluted FITC-and PE-labelled goat anti-mouse IgG (Abcam, Cambridge, UK). Then, the cells were washed with PBS, resuspended in 500 μL PBS, and subsequently characterized by flow cytometric analysis [32]. Viable lymphocytes were gated, and 15,000 events were analyzed for positive staining with FITC or PE per sample. Moreover, the remainding ducks from 1 week post-challenge were examined for CD4 and CD8 by the same procedure.

### 2.9. Statistical Analysis

Statistical analysis was performed using GraphPad Prism 6 (GraphPad Software, La Jolla, CA, USA). Paired student *t*-tests and one-way ANOVA were used to test differences between different groups. *p*-values < 0.05 were considered significant.

## 3. Results

### 3.1. Virulence and Sustainability of Recombinant DEV-ompH Virus in Tissues

**Figure 1:**
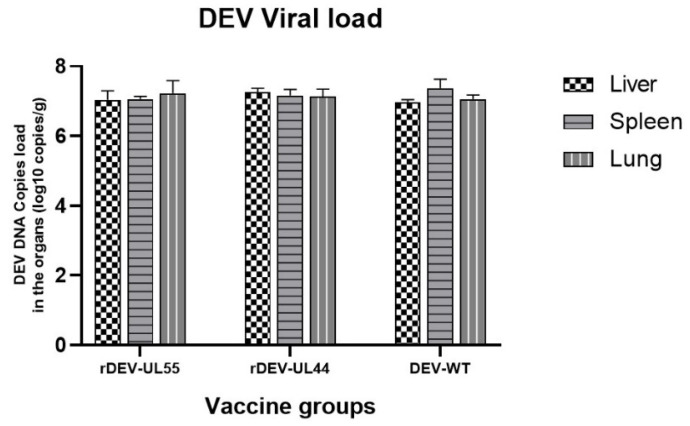
DEV viral load in organs infected with rDEV-OmpH-UL55, rDEV-OmpH-UL44 or DEV-WT. All the ducks in the three groups were humanely euthanized on day 7 post-inoculation, and their organs (liver, spleen and lungs) were obtained to determine virus titers using a one-step real-time PCR assay. (*p* < 0.05).

To investigate whether the insertion of the ompH gene affects DEV, the number of viruses sustained in tissues from ducks infected with rDEV-OmpH-UL55 and rDEV-OmpH-UL44 were determined. Ducks were vaccinated with 10^5^ PFU of rDEV-OmpH-UL55, rDEV-OmpH-UL44 and DEV-WT (n = 5 per group) intramuscularly. All the ducks remained healthy until 7 days. Then, the ducks were humanely euthanized, and organs (liver, spleen, lung) were collected to determine virus titers using a one-step real-time PCR assay. As shown in Figure 1, rDEV-OmpH-UL55 and rDEV-OmpH-UL44 could be detected in all tested tissues (liver, spleen, lungs) with high viral loads. There was no significant difference between DEV-WT and both recombinant DEV-ompH viral vectors, suggesting that the insertion of the ompH gene did not increase the virulence of the attenuated DEV vaccine strain and change the replication ability of DEV-WT in vivo.

### 3.2. The Serological Responses against DEV and P. multocida Strain X-73 Measured by ELISA

**Figure 2:**
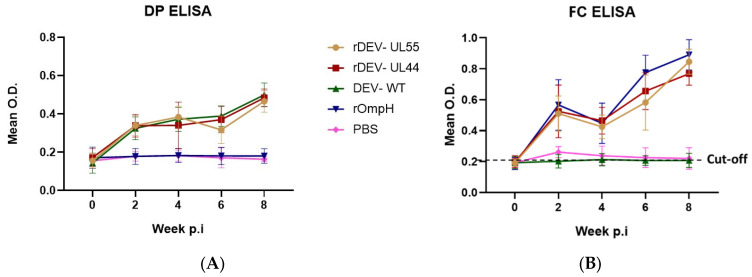
Examination of immunogenicity of rDEV-UL55 and rDEV-UL44 compared with DEV-WT, rOmpH and PBS post-immunization in ducks against DEV (**A**) and *P. multocida* X-73 (**B**) using ELISA.

To evaluate the duck antibody response of rDEV-UL55 & rDEV-UL44 against DEV, ducks were intramuscularly inoculated with different vaccines as described previously. Serum samples were collected every 2 weeks for 8 weeks post-vaccination (pv) from all the groups to monitor the serological antibodies against DEV using ELISA. As shown in Figure 2A, the mean antibody levels against DEV were above the cut-off, significantly higher than the rOmpH group and PBS control group. Moreover, there were no differences in antibody levels in both the rDEV-ompH groups and the DEV-WT group. Antibody levels in the PBS and rOmpH-inoculated groups were lower than the cut-off, considered negative throughout the experiment. These results indicate that the insertion of the ompH gene did not alter the immunogenicity of the DEV vaccine strain.

In order to investigate the duck antibody response of rDEV-UL55 and rDEV-UL44 against *P. multocida* strain X-73, all duck sera were analyzed at the same time point by ELISA. As shown in Figure 2B, the mean antibody levels against *P. multocida* in the rDEV-ompH-UL55, rDEV-ompH-UL44 and rOmpH groups were significantly above the cut-off level and were much higher than DEV-WT and PBS control groups. Similar trends in the antibody responses, with a dramatic increase in the levels, were observed in the rDEV-ompH-UL55-, rDEV-ompH-UL44- and rOmpH-inoculated groups. The strong antibody responses in these groups reached their peak 2 weeks post final immunization at the same level, while antibody responses in the DEV-WT and PBS control groups remained lower than the cut-off line during the entire experimental period. The qualitative analysis of the humoral responses demonstrates that ducks vaccinated with rDEV-ompH-UL55 and rDEV-ompH-UL44 did elicit humoral immune responses against DEV and *P. multocida* strain X-73 simultaneously.

### 3.3. Vaccine Efficacy against Lethal DEV and P. multocida Strain X-73 Challenge in Ducks

**Figure 3:**
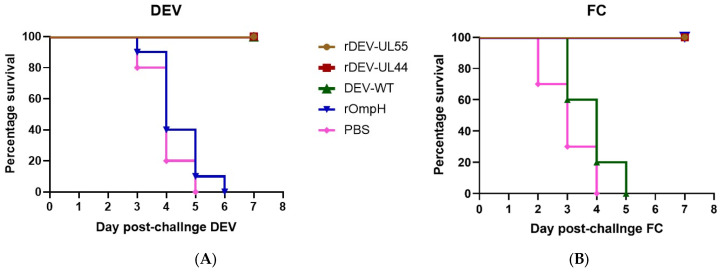
Protective efficacy of the rDEV-UL55 and rDEV-UL44 against virulent DEV (**A**) and *P. multocida* X-73 (**B**) challenge in ducks. Ducks were examined daily for 1 week after challenge.

Protection against virulent pathogens is the best criterion to assess the efficacy of vaccines. For this, animal experiments were carried out to test the clinical protective efficacy of the recombinant DEV-ompH vaccines. After challenging the ducks with a 100-fold 50% duck lethal dose (DLD_50_) of local virulent DEV strain by intramuscular injection, the ducks were observed for clinical signs for 7 days. All the immunized ducks in rDEV-ompH-UL55, rDEV-ompH-UL44 and DEV-WT groups exhibited no sign of clinical disease or any vaccine-induced overt pathology. In comparison, the ducks in the rOmpH and PBS control groups exhibited typical clinical signs of the duck plague starting from 3 days post-challenge, including listlessness, ruffled feathers, and anorexia, and ultimately succumbed to disseminated infection within 6 days pc (Figure 3A). No significant difference was observed in the protective efficacy between the rDEV-ompH vaccinated and DEV-WT groups against lethal DEV challenge. Moreover, autopsies were done on all dead ducks of the rOmpH and PBS group, and typical lesions on organs were taken for analysis of the viral load of DEV DNA by real-time PCR. It is not surprising in both groups that the challenged viruses were replicated efficiently in the liver, spleen, and lungs, with high viral loads ranging from 6.6 to 7.4 log10 50% egg infectious doses (log10EID_50_/g). Thus, data confirmed that the recombinant DEV simultaneously encoding the ompH gene did not appear to impair the protective ability of the attenuated DEV vaccine.

Next, we also examined whether both recombinant rDEV-ompH-UL55 and rDEV-ompH-UL44 would confer protection against *P. multocida* strain X-73 challenge. Following *P. multocida* 3.5 × 10^3^ PFU/mL infection, all the ducks immunized with both rDEV-ompH and rOmpH vaccines were completely protected and showed no clinical signs, with all ducks surviving during the 1-week observation period. In contrast, the ducks exposed to DEV-WT and PBS showed typical clinical signs of the fowl cholera disease with depression, ruffled feathers, loss of appetite, diarrhea, nasal and ocular discharge, and sudden death from 2–5 d pc (Figure 3B). Furthermore, bacterial isolation results from tissues showing gross lesions were positive for the isolation of *P. multocida* strain X-73 (data not shown). Taken together, these data demonstrate that both recombinant DEV-ompH-UL55 and DEV-ompH-UL44 vaccines immunization provided significant simultaneous protection against *P. multocida* as well as DEV challenge.

### 3.4. Detection of IFN-γ and IL-4 in Serum as the Cellular Response to Recombinant DEV-ompH-UL55 and DEV-ompH-UL44

**Figure 4:**
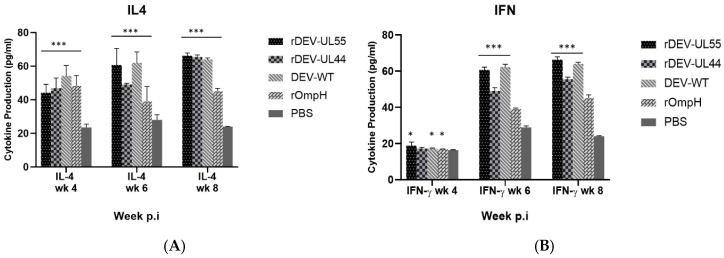
Cellular response to rDEV-UL55 & rDEV-UL44 vaccination. Serum IL-4 (**A**) and IFN-γ (**B**) cytokine levels in ducks were assessed before challenge at 4, 6, and 8 weeks post-immunization. Statistically significant differences are indicated by (***) *p*  <  0.001, (**) *p*  <  0.01, and (*) *p*  <  0.05 compared with the PBS control.

Duck sera from each group were collected for cytokine assay before challenge at 4, 6, and 8 weeks post-immunization (pi). Th1-type and Th2-type cytokine responses to the DEV were assessed by measuring the serum levels of IFN-γ and IL-4, respectively, using commercial duck IFN-γ and IL-4 sandwich ELISA. As shown in Figure 4A,B, respectively, higher levels of Th2-associated IL-4 and Th1-associated IFN-γ were observed in both rDEV-UL55 and rDEV-UL44-receiving groups compared to the PBS group (*p* < 0.001). Moreover, the IL-4 response of vaccine groups started higher than the control group at 4 weeks pi, but the levels of the IFN-γ response began to be higher at 6 weeks. These results illustrated the induction of cellular immune response. A comparison of the rDEV-ompH-UL55 to rDEV-ompH-UL44 vaccine showed that both recombinant vaccines were not significantly different in eliciting the cellular immune response via cytokine expression at 4, 6 and 8 weeks post-vaccination (*p* < 0.05).

**Figure 5:**
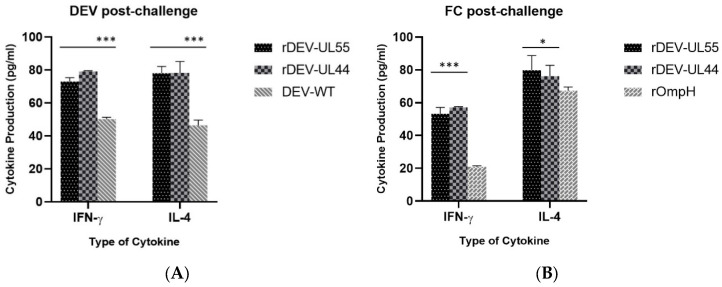
Cellular response to rDEV-UL55 and rDEV-UL44 post-challenge with DEV (**A**) or *P. multocida* (**B**). Serum IFN-γ and IL-4 cytokine levels in surviving ducks were analyzed at 7 days post-challenge. Statistically significant differences are indicated by (***) *p*  <  0.001 and (*) *p*  <  0.05 comparing the recombinant DEV-OmpHs with the DEV-WT or rOmpH group.

Furthermore, the cytokine levels in the vaccinated ducks were determined in different groups post-challenge with DEV and *P. multocida*, respectively (Figure 5). The cellular immune response measured by IFN-γ and IL-4 levels (Figure 5A,B) showed that ducks immunized with both rDEV-ompH constructs induced significantly higher immunity than in the positive groups immunized with DEV-WT or rOmpH (*p* < 0.001). The levels of IFN-γ and IL-4 between rDEV-ompH-UL55 or rDEV-ompH-UL44 post-challenge DEV and *P. multocida* were not significantly different (*p* < 0.05). The trend of IFN-γ and IL-4 levels indicate that targeting ligands may enhance the production of memory lymphocytes and humoral immunity.

### 3.5. The Cellular Response to Recombinant DEV-ompH-UL55 and DEV-ompH-UL44 by In Vitro Lymphocyte Proliferation Assay (LPA)

**Figure 6:**
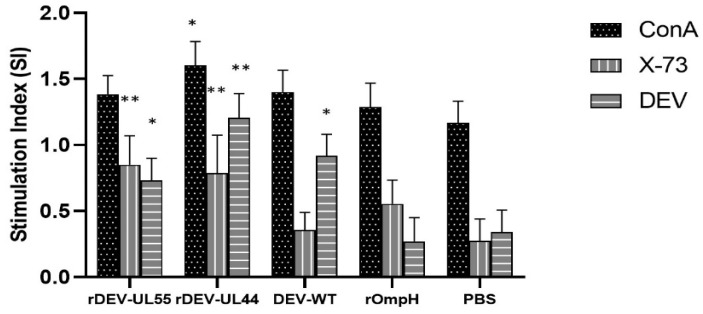
Peripheral blood T-lymphocyte proliferation assays were evaluated by MTT assay at 8 weeks post-immunization. Data are shown as the SI mean  ±  SD. Statistically significant differences are indicated by (**) *p*  <  0.01 and (*) *p*  <  0.05 compared with the PBS control.

Cellular immune response was further evaluated by determining the capability of the proliferative response of duck peripheral mononuclear cells against the purified heat extract protein (HE) of *P. multocida* strain X-73 or DEV and ConA. As shown in Figure 6, a significantly enhanced T-cell proliferative response to HE or DEV stimulation was observed in the groups immunized with both rDEV-ompH vaccines at levels higher than DEV-WT and rOmpH groups, respectively (*p* < 0.01). No significant measurement of T-cell proliferation between rDEV-ompH-UL55 and rDEV-ompH-UL44 to HE stimulation was detected. DEV stimulation resulted in a significantly higher response of rDEV-ompH-UL44 compared to rDEV-ompH-UL55 (*p* < 0.05). The control group that received PBS buffer did not respond to both stimulators. Ducks in all groups responded similarly to stimulation with positive control ConA. These results indicate that both rDEV-ompH-UL55 and rDEV-ompH-UL44 constructs can elicit a robust cellular immune response in ducks.

### 3.6. Analysis of CD4+ and CD8+ T Lymphocytes in Peripheral Blood

**Figure 7:**
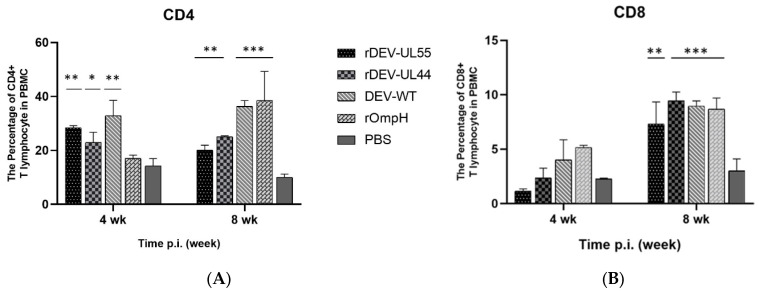
Analysis of CD4+ (**A**) and CD8+ (**B**) T lymphocytes in peripheral blood using flow cytometry at 4 weeks and 8 weeks post-vaccination. Statistically significant differences are indicated by (***) *p*  <  0.001, (**) *p*  <  0.01, and (*) *p*  <  0.05 compared with the PBS control.

To determine the population of CD4+ and CD8+ T lymphocytes in PBMCs, the peripheral blood lymphocytes were collected, processed and analyzed by flow cytometry at 4 and 8 weeks post-immunization. Figure 7 shows the percentages of CD4+ (Figure 7A) and CD8+ (Figure 7B) T lymphocytes in the rDEV-ompH-UL55 and rDEV-ompH-UL44 immunized groups were significantly higher than in the negative control group (*p* < 0.05). At 8 weeks post-vaccination, the CD4+ percentage of both rDEV-ompH groups was lower than the DEV-WT and rOmpH groups. At the same time, the CD8+ percentage of the rDEV-ompH-UL44 group shows a similar level to DEV-WT and rOmpH groups. Similarly, the CD8+ percentage of the rDEV-UL55 group was not significantly lower than the other vaccinated groups (*p* < 0.05) (Figure 7B). The total cellular response increased and remained high at 4 weeks and at 8 weeks post-vaccination. These results indicated that both CD4+ and CD8+ T lymphocytes from the vaccinated group reached higher levels than those in the negative control group (*p* < 0.05), while the numbers of CD4+ T lymphocytes were larger than those of CD8+ T lymphocytes at the same time points.

**Figure 8:**
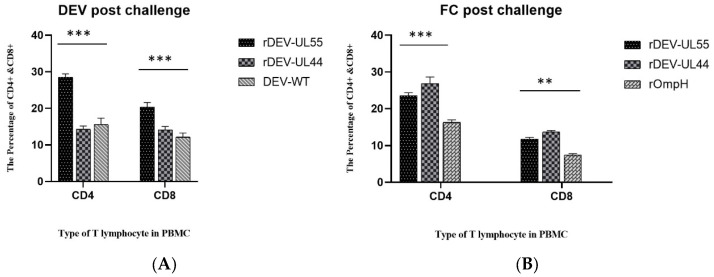
Levels of CD4+ and CD8+ response in survived ducks were analyzed at 7 days post-challenge with lethal DEV (**A**) and *P. multocida* X-73 (**B**) in different groups. Statistically significant differences are indicated by (***) *p*  <  0.001, (**) *p*  <  0.01 comparing the recombinant DEV-OmpHs with the DEV-WT or rOmpH group.

Moreover, the CD4+ and CD8+ T lymphocytes response of the ducks that survived the challenge with DEV and fowl cholera were continually measured (Figure 8). After the DEV challenge (Figure 8A), the CD4+ and CD8+ T lymphocytes of the rDEV-ompH-UL55 group displayed the highest values, while values of the rDEV-ompH-UL44 group were maintained at a similar level to the DEV-WT group (*p* < 0.01). Conversely, post-FC challenge (Figure 8B), the rDEV-ompH-UL44 group exhibited the highest values for both CD4+ and CD8+ T lymphocytes, followed by rDEV-ompH-UL55 and rOmpH, consecutively (*p* < 0.05). This result indicates precisely that both rDEV-ompH vaccines enhanced T helper cell proliferation and induced high levels of cellular immune response post-infection.

## 4. Discussion

Recently, increasing evidence has shown that novel viral vector vaccines have the potential to greatly improve poultry disease control through the offering of simple, convenient, cost-effective, safe and efficacious means of vaccine delivery capable of the induction of long-lasting humoral, mucosal and cell-mediated immunity [33]. Fowlpox virus, Turkey herpesvirus and Adenovirus are the major vectors currently used for the construction of recombinant vaccines for poultry. With the use of those vectors, more than 15 recombinant viral vector vaccines against Newcastle disease (ND), infectious laryngotracheitis [34], infectious bursal disease (IBD), avian influenza (AI), and *Mycoplasma gallisepticum* (MG) have been developed and are commercially available [35]. More recently, the first commercial trivalent vector vaccine has enabled to vaccinate poultry against up to three important pathogens (HVT + ND + IBD or HVT  +  IBD  +  ILT) with a single in ovo administration. All these viral vector vaccines offer the potential for safe, convenient and rapid mass delivery using several routes of administration [36]. Additionally, attenuated DEV with glycoprotein C (gC) deletion may be exploited for the development of vectored vaccine candidates [37]. From our previous study, we successfully generated two recombinant DEV-ompH-UL55 and DEV-ompH-UL44 vaccines with the ompH genes of *P. multocida* inserted into the genome of the DEV vaccine strain at the UL55-LORF11 and UL44-44.5 intergenic regions, respectively, using the NHEJ-CRISPR-Cas9 system [27]. To the best of our knowledge, that was the first time the CRISPR/Cas9 system has been applied to insert a highly immunogenic gene from bacteria into the DEV genome rapidly and efficiently. However, there has been little research on the immune responses to and the challenge results of recombinant DEV vaccines using CRISPR/Cas9. The result of this study performed in vivo confirmed the finding of our previous recombinant DEV vaccine study in vitro, showing that recombinant vaccines are safe, stable, and effective bivalent vaccines.

At present, an ELISA developed using the whole DEV virion usually acts as an antigen for the detection of antibodies against DEV in an indirect ELISA assay [38]. Our previous study demonstrated that there was no cross-reaction between antisera against DEV-*P. multocida* detected by ELISA [8]. IgG is the main antibody found in blood and mucosal surfaces against viruses and bacteria [39]. In this study, the rDEV-ompH-UL55 and rDEV-ompH-UL44 vaccines enhanced the production of IgG antibodies against DEV and *P. multocida*. An especially robust antibody response was observed 14 days after the booster vaccination. The results indicated that both rDEV-ompH vaccines were safe and induced specific anti-DEV and anti-*P. multocida* humoral immune responses, which coincided with previous studies [29,32,40,41,42]. A previous study with a DEV-vectored H5 vaccine, rDEV-us78HA and rDEV-H5-UL55, constructed with the cosmid and BAC system, respectively, provided strong 100% protection against both duck plague and highly pathogenic AIV H5N1 despite eliciting a weak hemagglutination inhibition (HI) titer in commercial ducks [22,43,44].

In the immune responses to infection or vaccination, the T-cell response is indispensable for effective immunity. IL-4 plays a critical role in regulating the behavior of hematopoietic cells. In T-cells, IL-4 acts as a co-stimulant of cell growth and controls Th2 polarization. IFN-γ is a key cytokine produced primarily by T cells and natural killer cells, which can facilitate the host’s defense against intracellular pathogens [45]. In this study, the concentrations of IFN-γ and IL-4 in the ducklings vaccinated with both rDEV-ompHs were highest at 8 weeks after primary immunization. Moreover, post-challenge with DEV and *P. multocida*, the level of specific IL-4 and IFN-γ of both rDEV-OmpH groups were at their peaks and significantly higher than those of the DEV-WT or rOmpH vaccine groups. These results suggested that rDEV-OmpHs could stimulate a strong T cell response against DEV and *P. multocida* and could fortify protection against both pathogens.

A basic aspect of protective immunity is effective cooperation between T and B cells [46]. CD4+ T cells are necessary to generate a CD8+ CTL response, and both T lymphocytes play an important role in viral clearance responses after infection with herpesviruses [47]. Additionally, CD4+ T cells have positive feedback with humoral immunity [48]. From our observations, the levels of both CD4+ and CD8+ T lymphocytes increased significantly after immunization with both the rDEV-ompH vaccines. The highest numbers of CD4+ T cells were found earlier than CD8+ T cells, and the CD4+ T cell population increased greatly, but CD8+ T cells increased to a lesser extent in immunized ducks. This might be due to the fact that CD4+ T lymphocytes are the predominant factor in cellular immune responses [49,50]. Nevertheless, the substantial CD4+ and CD8+ T cell responses in inoculated ducks remained high post-challenge. These results demonstrate that the rDEV-ompH-UL55 and rDEV-ompH-UL44 vaccines could activate T lymphocytes and stimulate ducks to produce cell-mediated immune responses after vaccination. The efficient innate immune response is essential for the initial detection of invading viruses and subsequent activation of adaptive immunity against the virus [51].

## 5. Conclusions

Overall, this study provides valuable information for understanding the immune responses to the rDEV-ompH-UL55 and rDEV-ompH-UL44 bivalent vaccines against DEV and *P. multocida* in ducks. This investigation has indicated that rDEV-ompH-UL55 and rDEV-ompH-UL44 generated by the CRISPR-Cas9 Cre-Lox system has the potential to induce antibody production and lymphocyte proliferation and increase the numbers of CD4+ and CD8+ T cells in PBMCs. More importantly, both bivalent vaccines conferred full simultaneous protection against the lethal DEV and *P. multocida* challenge in ducks. Finally, by targeting multiple diseases with a single vaccine, there should be opportunities to reduce the cost and attain greater farmer acceptability, resulting in increased confidence in the uptake of the vaccine.

## Figures and Tables

**Table 1:** Vaccine immunization and challenge exposure in duck.

Group	Vaccination Formulation	Challenge Exposure (IM) Duck/Group	
		Virulent Local Type DEV (100-fold DLD_50_)	*P. multocida* X-73 (3.5 × 10^3^ CFU/mL)
1	rDEV-Omp-UL55 10 ^5^ PFU ^a^	10	10
2	rDEV-Omp-UL44 10 ^5^ PFU ^a^	10	10
3	DEV-WT 10 ^3.5^ PFU ^b^	10	10
4	rOmpH 100 µg/mL	10	10
5	PBS	10	10
Total		100	

^a^ Recommended dose for the DEV vaccine. ^b^ Dose for the commercial DEV vaccine.

## Data Availability

All data in this study have been included in the manuscript.
